# Development and evaluation of a novel fast broad-range PCR and sequencing assay (FBR-PCR/S) using dual priming oligonucleotides targeting the ITS/LSU gene regions for rapid diagnosis of invasive fungal diseases: multi-year experience in a large Canadian healthcare zone

**DOI:** 10.1186/s12879-022-07356-9

**Published:** 2022-04-13

**Authors:** B. Chow, M. Groeschel, J. Carson, T. Griener, D. L. Church

**Affiliations:** 1 Department of Pathology and Laboratory Medicine, Cummings School of Medicine, University of Calgary, 3330 Hospital Dr. NW, Calgary, AB T2N 4B1 Canada; 2 Department of Medicine, Cummings School of Medicine, University of Calgary, 3330 Hospital Dr. NW, Calgary, AB T2N 4B1 Canada; 3 Calgary Laboratory Services (Now Alberta Precision Laboratories), 9-3535 Research Rd NW, Calgary, AB T2L 2K8 Canada

**Keywords:** Fungal infection, Broad-range fungal PCR, Sequencing, Molecular diagnosis

## Abstract

**Background:**

This study evaluated the performance of a novel fast broad range PCR and sequencing (FBR-PCR/S) assay for the improved diagnosis of invasive fungal disease (IFD) in high-risk patients in a large Canadian healthcare region.

**Methods:**

A total of 114 clinical specimens (CS) including bronchoalveolar lavages (BALs) were prospectively tested from 107 patients over a 2-year period. Contrived BALs (n = 33) inoculated with known fungi pathogens were also tested to increase diversity. Patient characteristics, fungal stain and culture results were collected from the laboratory information system. Dual-priming oligonucleotide (DPO) primers targeted to the internal transcribed spacer (ITS) (~ 350 bp) and large subunit (LSU) (~ 550 bp) gene regions were used to perform FBR-PCR/S assays on extracted BALs/CS. The performance of the molecular test was evaluated against standard microbiological methods and clinical review for the presence of IFD.

**Results:**

The 107 patients were predominantly male (67, 62.6%) with a mean age of 59 years (range = 0–85 years): 74 (69.2%) patients had at least one underlying comorbidity: 19 (34.5%) had confirmed and 12 (21.8%) had probable IFD. Culture recovered 66 fungal isolates from 55 BALs/CS with *Candida* spp. and *Aspergillus* spp. being most common. For BALs, the molecular assay vs. standard methods had sensitivity, specificity, positive predictive value (PPV) and negative predictive value (NPV), and efficiency of 88.5% vs.100%, 100% vs. 61.1%, 100% vs. 88.5%, 61.1% vs. 100%, and 90.2% for both. For other CS, the molecular assay had similar performance to standard methods with sensitivity, specificity, PPV, NPV and efficiency of 66.7%, 87.0%, 66.7%, 87.0% and 81.3% for both methods. Both methods also performed similarly, regardless of whether CS stain/microscopy showed yeast/fungal elements. FBR-PCR/S assays results were reported in ~ 8 h compared to fungal cultures that took between 4 and 6 weeks.

**Conclusions:**

Rapid molecular testing compared to standard methods have equivalent diagnostic efficiency but improves clinical utility by reporting a rapid species-level identification the same dayshift (~ 8 h).

## Background

Invasive fungal disease (IFD) has increased significantly in the last few decades due to the expansion of patients with acquired immunosuppression [1–4]. IFD results in increased morbidity and mortality and higher healthcare costs [5–10]. Delayed diagnosis is associated with poor clinical outcomes because appropriate treatment measures are not promptly started [1, 11, 12]. However, IFD is often difficult to diagnose because clinical and radiographic findings are non-specific [4]. Traditional microbiological methods such as fungal culture also have low sensitivity ranging from 30 to 60% [13, 14], and the lack of concordance between histopathology and cytology examination and culture is well documented [15–17].

Molecular methods including broad-range PCR followed by sequencing are increasingly being used for definitive identification of fungal pathogens and improved diagnosis of IFD [4, 14, 18]. The aim of this study was to identify unique primer candidates for broad-range amplification of the fungal internal transcribed spacer (ITS) and large subunit (LSU) gene regions to use in a fast PCR/sequencing assay that could be rapidly completed in a clinical laboratory. Prior studies evaluating panfungal PCR assays have relied on conventional primers targeted to one or more regions of the fungal multi-copy ribosomal RNA (rRNA) such as 18S rRNA, D1and D2 regions of 28 s rRNA, 5.8S rRNA, and internal transcribed spacers 1 and 2 (ITS1 and ITS2) with variable success [19–28]. We designed a new primer pair based on the dual priming oligonucleotide (DPO) principle because of our success with this approach in previously implementing a broad-range 16S rRNA PCR/sequencing assay with robust sensitivity and improved specificity due to elimination of cross-reactivity with human material [29]. A DPO consists of two functional segments with distinct annealing properties connected by five consecutive deoxyinosine bases or a poly (I) linker; (1) a 5’ segment (18–25 bp) allows for stable positioning and annealing of the primer, and (2) a shorter segment (6–12 bp) that will only bind if there is stable annealing of the 5’ end to ensure target-specific extension [30] Diagnostic performance of our novel fungal FBR-PCR/S assay was compared to standard microbiological methods already in use in our laboratory (i.e., morphology, fungal culture with identification using matrix-assisted laser desorption ionization-time of flight mass spectrometry (MALDI-TOF MS) and PCR/sequencing using conventional ITS 1/2 universal primers). A recommended algorithm is provided for clinical laboratories to allow reporting the same day by efficient integration of technologists’ workflow for simultaneously performing bacterial and fungal broad-range PCR/cycle sequencing assays within a standard ~ 8 h dayshift.

## Materials and methods

### Patients and clinical specimens

Patients with and without suspected non-invasive and IFD were prospectively enrolled over a 2-year period (2016–18) from the Calgary Zone, Alberta Health Services (AHS) based on combined concern of the consulting Infectious Diseases physician for IFD, and the results of microbiological analyses of clinical specimens. Cases were categorized as having proven probable or possible IFD or no fungal disease based on consensus definitions recently published by the European Organization for Research and Treatment of Cancer and the Mycoses Study Group Education and Research Consortium (EORTC/MSGERC) following clinical review by medical microbiologists (MG and JC) and an infectious diseases specialist (DLC) [4].

Sterile fluid and tissue specimens were enrolled by the microbiology laboratory (Clinical Section of Microbiology, Calgary Laboratory Services (CLS; now Alberta Precision Laboratories) after quality approval by a medical microbiologist (DLC/TG/MG). Study specimens were stored at − 80 to − 86 °C before analyses. Stored clinical specimens and contrived bronchoalveolar lavages (BALs) were used for pre-clinical validation of the molecular assay. Contrived BAL specimens were prepared to simulate a heavily infected sample (up to 35 ng DNA). DNA was extracted from spent fungal-negative BAL specimens inoculated with known pathogens (n = 33) obtained from our reference mycology laboratory [Provincial Laboratory Northern Alberta (PLNA), Edmonton, AB] including: *Aspergillus lentulus* (n = 1), *A. terreus* (n = 3), *A. flavus* (n = 3); *Absidia corymbifera* (n = 3); *Fonsecaea pedrosoi* (n = 2); *Fusarium solani* (n = 2), *F. proliferatum* (n = 1); *Cladosporium carrionii* (n = 2) and *Cladosporium* spp. (n = 1); *Cunninghamella bertholletiae* (n = 2) and one undetermined *Cunninghamella* spp.; *Rhizopus aarhizus* (n = 1), *R. microsporus* (n = 1), *R. stolonifera* (n = 1), and three undetermined *Rhizomucor* spp.; *Trichosponon asahii* (n = 1) and one undetermined *Trichosporon* spp.; and *Malassezia furfur* (n = 2), *M. pachydermatis* (n = 1).

### Microbiological analyses

Clinical specimens were analyzed by standard microscopic examination and fungal culture methods. Yeast isolates were identified by microscopic examination, and matrix-assisted laser desorption/ionization-time-of-flight mass spectrometry (MALDI-TOF MS) (Vitek MS, bioMérieux, Laval, Quebec). Molds were identified using colony morphology, microscopic examination and conventional PCR using the internal transcribed spacer or ITS gene regions (including universal ITS1 and ITS2) as previously described by White and colleagues [31]. Identification of fungal isolates provided by the PLNA reference laboratory was confirmed using the MicroSEQ™ D2 rDNA Fungal PCR and Sequencing Kit (Applied Biosystems, Thermo Fisher Scientific).

### Molecular methods

- Controls

**Fig. 1:**
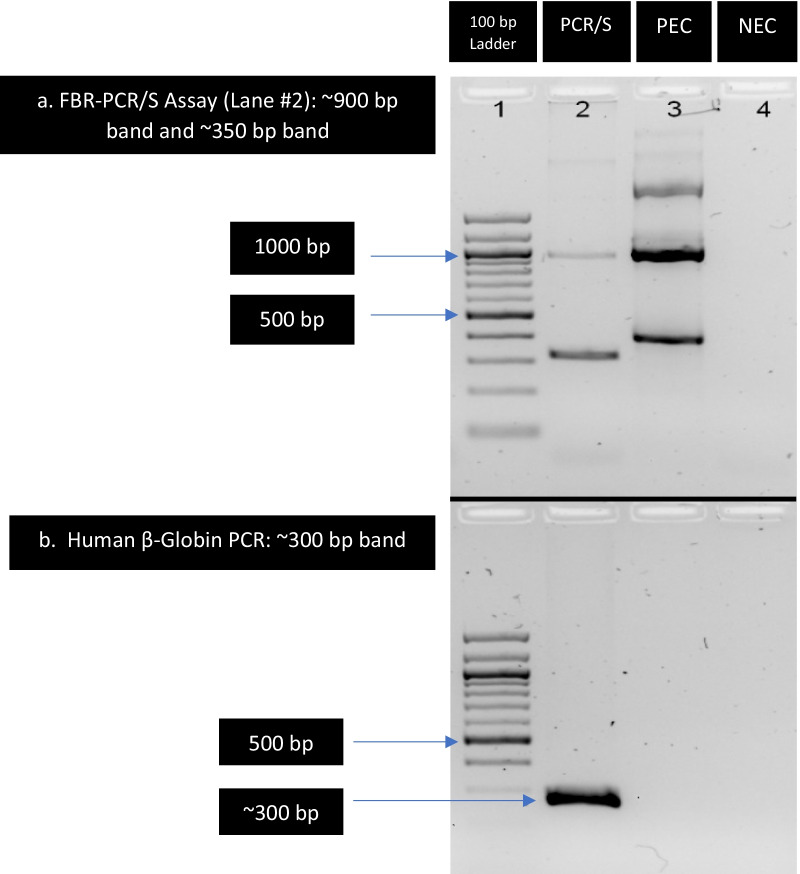
Electrophoresis Gel Image of FBR-PCR/S Assay^a^. ^a^FBR-PCR/S assay gel image: Lane 1: 100 bp ladder; Lane 2: Clinical sample; Lane 3: Positive Extraction Control (PEC) (*S. cerevisiae*); Lane 4: Negative Extraction Control (NEC). ^a^ Top Panel: Fungal Broad-range (FBR) Panfungal PCR targeting ITS and LSU gene regions. FBR PCR shows two bands, ~ 350 bp ITS band and ~ 900 bp band corresponding to combined amplification of ITS (~ 350 bp) and LSU (~ 600 bp) targets. ^b^ Lower Panel: human β-globin PCR. Human clinical sample shows a strong ~ 300 bp band, indicating successful DNA extraction

A clinical isolate of *Saccharomyces cerevisiae* positive for both ITS2 and LSU targets was used as the positive control for all molecular procedures (See Fig. 1—Positive Extraction control or PEC). The negative extraction control (i.e., extraction reagents only; NEC) was processed and extracted alongside all clinical and contrived specimens throughout all FBR-PCR/S assay procedures (See Fig. 1—NEC).

b. DNA Extraction

Fungal isolates obtained from the reference laboratory were extracted in TE buffer using glass bead beating. Nucleic acid DNA concentration was determined by a Nanodrop spectrophotometer (Thermo-Fisher Scientific, Mississauga, ON). A total of 500 ng DNA was eluted into 100 µL of TE buffer giving a final template concentration of 5 ng/µL to give reliable detection. Contrived specimens (n = 33) consisted of 500 ng reference isolate DNA added to 400 µL of spent culture-negative BAL fluid from spent clinical specimens whose clinical analyses were complete. Clinical and contrived specimens were extracted using the QIAmp UCP Pathogen Mini Kit (Canada-QIAGEN, Toronto, CA) according to the manufacturer’s protocol. Tissues had an extended Proteinase K incubation time, otherwise both tissue and fluid protocols were the same. Tissue specimens (2–4 mm^3^) were finely minced with a sterile scalpel, transferred to a sterile 1.5 mL microcentrifuge tube, re-suspended in 400 µL Buffer ATL and 40 µL Proteinase K, vortexed, and incubated at 56 °C in a 1000 rpm Eppendorf thermomixer for 1 h until digested. Sterile fluid specimens (at least 400 µL) were placed into a sterile 1.5 mL microcentrifuge tube, centrifuged, supernatant discarded, cell pellet re-suspended in Buffer ATL, Proteinase K, vortexed, and incubated at 56 °C in a 1000 rpm Eppendorf thermomixer for a minimum of 10 min. Purified, eluted DNA was stored at − 20 °C until use.

c. FBR-PCR/S Assay

DPO primers (26, 27) targeted towards the Internal Transcribed Spacer (ITS) regions and the Large Subunit (LSU) of the nuclear ribosomal RNA (rRNA) gene complex were designed by the investigators (BC and DLC) after multisequence alignment of several hundred GenBank sequences of multiple genera to identify candidate conserved regions. DPO primers were purchased from Exiqon (Woburn, MA). All other primers were purchased from Integrated DNA Technologies (IDT, Coralville, Iowa). FBR-PCR used a forward primer [ITS3DPO_F3: 5′ CAT CGA TGA AGA RCG YA-I-I-I-I-I-I-TGCGA 3′ (I = deoxyinosine; R = A/G, Y = C/T)], and two reverse primers, for ITS detection [ITS4DPO_R5: 5′ TAT TGA TAT GCK TAA-I-I-I-I-I-G CGG GT 3′ (K = G/T), and LSU detection [LSUDPO3_R: 5′ GAC TCC TTG GTC CGT-III-II-AAG AC 3′. PCR for human-β-globin gene was performed in parallel as a control using β-glob-F [GAAGAGCCAAGGACAGGTAC] and β-glob-PC04R [CAACTTCATCCACGTTCACC] in a final concentration of 0.3 µM.

Fast-PCR was set up with the Molyzm 16S basic (Molzym, Bremen, Germany) kit reagents. The 30 µL reaction contained 7 µL of template DNA and final concentration of 0.3 µM ITS3DPO_F3 forward primer and 0.2 µM each of ITS4DPO_R5 and LSUDPO3_R reverse primer. FBR-PCR was performed on a Veriti thermocycler (Life Technologies, Carlsbad, CA) under the following cycling conditions: 5 min. initial denaturation at 95 °C, followed by 35 cycles of 94 °C for 10 s, 54 °C for 15 s and 72 °C for 25 s, with a final extension of 72 °C for 5 min. PCR product was electrophoresed on a 1.5% agarose gel containing SYBRsafe (Life Technologies). During the PCR reaction, the ITS3DPO_F3/ITS4DPO-R5 F/R primer pair amplify a ~ 350 bp ITS amplicon, whereas the ITS3DPO_F3/LSUDPO_3R F/R primer pair amplify the ITS region (~ 350 bp) plus ~ 500–600 bp of the LSU region. The ~ 900 bp amplicon therefore represented a combined ITS/LSU fragment. Agarose gel electrophoresis confirmed the amplification of fungal DNA: PCR products displaying a band in the expected ~ 350 bp region for ITS, and ~ 900 bp region for ITS/LSU were then purified by Exo-SAP-it (Affymetrix, Santa Clara, CA) (Fig. 1).

Assay level of detection (LoD) was determined using two well characterized isolates; *Aspergillus brasiliensis* (ATCC 16404) and *Candida albicans* (ATCC 10231). Briefly, DNA was extracted in a 1.5 mL microfuge containing ~ 0.2 g of zirconium beads (BioSpec) and 10 mM Tris-1 mM EDTA pH 8.0 buffer and subsequently boiled at 100 °C for 10 min. to inactivate the organisms. Bead beating at 5 m/s for 120 s occurred in a Beadmill 4 instrument (Fisher Scientific) before centrifugation to pellet the debris and transfer of the supernatant to a fresh tube. A Nanodrop spectrophotometer (ThermoFisher) was used to measure the DNA concentration before subsequent 1/100 dilution with nuclease-free water to make a working solution. Copy number equivalents for each organism were calculated by using the genome size information by ATCC. LoD was then determined by a twofold serial dilution series of contrived negative BAL prepared with inoculation of either known amounts of ATCC 10231 or ATCC 16404 as follows: (1) “4 N” (4 × 10^5 copies/mL), (2) “2 N” (2 × 10^5^ copies/mL), (3) “D2” (5 × 10^4^ copies/mL), and 4) “D8” (1.25 × 10^4^ copies/mL). DNA was extracted from 400 μL of each contrived BAL specimen in the dilution series with the UV-irradiated Qiagen DNA mini kit (Qiagen) performed in triplicate. FBR-PCR was performed on each contrived BAL specimen as outlined above. Agarose gel electrophoresis confirmed the amplification of fungal DNA: PCR products displaying a band in the expected ~ 350 bp region for ITS, and ~ 900 bp region for ITS/LSU were then purified by Exo-SAP-it (Affymetrix, Santa Clara, CA). The dilution series established the LoD for the FBR-PCR assay for both *C. albicans* and *A. brasiliensis* as < 360 to > 175 copies/mL.

Molecular identification of the ITS and LSU rDNA product(s) was done by Sanger sequencing of the ITS and/or LSU rDNA product using BigDye Terminator v1.1 Cycle Sequencing Kit (Life Technologies) on an ABI Prism 3500XL sequencer (Life Technologies). The ITS3DPO_F3/ITS4DPO-R5 F/R primer pair was used to sequence the ITS region and the LSU-Fseq [AGTARCGGCGAGTGAAG]/ LSUDPO3_R F/R primer pair were used to sequence the LSU region. A BLAST search against the IDNS Fungal database (SmartGene IDNS, Lausanne, Switzerland) provided a definitive identification of the organism to the genus- or species-level using the identify scores outlined by the Clinical Laboratory Standards Institute, Approved Guidelines MM-18 [32].

### Data analysis

Data were entered into a Microsoft Excel spreadsheet (MS Office 2016) and analyzed according to standard descriptive statistics. A 2 X 2 contingency table was used to calculate the sensitivity, specificity, positive and negative predictive values were calculated against internationally recognized diagnostic criteria for the presence of IFD [4]. FBR-PCR/S performance was calculated against standard methods (i.e., fungal culture on all samples followed by identification of yeasts by morphology/MALDI-TOF MS and molds by morphology/conventional PCR targeted to the ITS1 and ITS 2 gene regions. Invalid FBR-PCR/S results were defined as a weakly positive electrophoresis band in either of the ITS/LSU fungal targets with no quality sequence subsequently obtained. In patients with confirmed, probable or possible IFD the following performance criteria were assigned for the FBR-PCR/S assay: (1) a true positive result agreed with that of standard methods, (2) a false-negative or false-positive result was considered discordant with standard methods, and in patients with no evidence of IFD, a true negative result agreed with standard methods. Resolution of discordant results occurred by repeat FBR-PCR/S testing, repeat PCR testing using conventional PCR targeted to the ITS1 and ITS 2 gene regions, and clinical review.

## Results

### Patient characteristics and specimens

A total of 107 enrolled patients were predominantly male (67, 62.6%), had a mean age of 59 years (range = 0–85 years) with no significant age difference according to gender. Comorbidities in five patients were unknown due to missing data. Most patients (74/107, 69.2%) had at least one underlying comorbidity that predisposed them to IFD including diabetes mellitus (18/74, 24.3%), solid-organ malignancy/tumour (18/74, 24.3%), immunosuppressive therapy for non-malignant conditions (13/74, 17.6%), hematologic malignancy (12/74, 16.2%), hematopoietic stem cell transplant (HSCT) (5/74, 6.7%), HIV/AIDs (4/74, 5.4%), and end-stage renal disease (2/74, 2.7%). A total of 54/74 (73%) had clinical evidence for IFD including 19 patients (17.8%) with confirmed IFD, 12 (11.2%) with probable IFD, and 27 (%) with possible IFD. True positive molecular tests were found in a third of patients (15/54, 27.8%) with confirmed or probable IFD.

A total of 114 clinical specimens were tested from these patients including 39 (34.2%) BALs and 75 (65.8%) other types of sterile fluids and tissues; 7 patients had ≥ 2 specimens tested (Table 1). BALs and other pulmonary specimens (lung/bronchial/pleural aspirates or fluids) (n = 51, 44.7%) were the most tested sterile fluids. A wide range of different tissue types were tested representing the disseminated nature of IFD. A total of 55 (48.2%) specimens had yeast/fungi recovered by standard methods. Twenty (17.5%) specimens only had bacterial cultures done because yeast/fungal culture was not initially ordered—most of these specimens (n = 16, 80%) had negative Gram and CW stains and bacterial cultures, but 1 BAL and 2 abdominal fluid specimens grew *Candida albicans* (despite negative CW), 1 abdominal fluid showed yeast in the Gram stain and grew *C. albicans*, and 1 sinus aspirate grew *Aspergillus fumigatus*.

**Table 1:** Clinical specimens tested in validation of broad range fungal PCR/sequencing assay

Fungal culture^a^	Bronchoalveolar lavages (BALs)	Lung/Bronchial/Pleural	Cerebrospinal fluids (CSFs)	Other sterile fluids^b^	Other sterile tissues^c^	Total
Positive	34	3	2	9	7	55
Negative	5	6	3	9	16	39
Not Ordered	0	3	9	7	1	20
Total	39 (34.2%)	12 (10.5%)	14 (12.3%)	25 (22%)	24 (21%)	114

^a^Specimens where fungal culture was not ordered but bacterial cultures grew yeast/fungi were counted as positive

^b^No fungal culture was done on a CSF that tested negative for Cryptococcal antigen. Includes peritoneal/dialysates (n = 6), synovial/spine disc (n = 5), abdominal (n = 5), sinus/nose aspirate (n = 4), liver abscess (n = 3), brain/subdural (n = 1) and periorbital (n = 1)

^c^Includes heart (n = 6), brain (2), shoulder/hip membrane (n = 4), spine/vertebra (n = 2), bone foot/mandible (n = 2), mediastinal lymph node (n = 2), skin biopsy (n = 2), neck (n = 1), cheek (n = 1), parotid gland (n = 1)

### Fungi identified from contrived and clinical specimens

The FBR-PCR/S assay accurately identified 32/33 (97%) of the yeast/fungi inoculated into the contrived BAL specimens except for one specimen containing *A. terreus* (See Methods). Another sixty-six fungal isolates were recovered from fifty-five clinical specimens. *Candida* spp. (n = 36, 54.5%) [*C. albicans* (n = 18), *C. dublinensis* (n = 4), *C. glabrata* (n = 5), *C. kefyr* (n = 3), *C. krusei* (n = 2), *C. parapsilosis* (n = 1) and *C. tropicalis* (n = 2)] and *Aspergillus* spp. (n = 14, 16.7%) [*A. flavus* (n = 2), A, *fumigatus* (n = 7), *A. terreus* (n = 1), *A. niger* (n = 1), and 3 other *Aspergillus* spp.] was the most identified species. Other fungal species identified included *Alternaria* spp. (n = 1), *Coccidioides immitis* (n = 2), *Cryptococcus neoformans* (n = 1), *Exophilia dermatiditis* (n = 1), *Fonsecaea* spp. (n = 1), *Fusarium merismoides* (n = 1), *Histoplasma capsulatum* (n = 1), *Penicillium* spp. (n = 2), *Pseudallescheria boydii* complex (n = 2), *Trichophyton rubrum* (n = 1) and *Rhizopus oryzae* (n = 3). One BAL sample was also PCR positive for *Pneumocystis jirovecii* using specific PCR primers.

### Resolution of discrepant results

Discordant results were initially observed in 30 clinical specimens including 16 BALs and 14 other types of clinical specimens. Tables 2, 3 details the resolution of discrepant results. Of the 30 discordant results, 19 (63.3%) specimens [BALs (n = 10) and other clinical specimens (n = 9)] were resolved in favour of the molecular assay results (Table 2), while 11(36.7%) specimens [BALs (n = 6) and other clinical specimens (n = 5)] were resolved in favour of standard methods (Table 3). BALs were prone to contamination from patient’s airway colonization with *Candida* spp. and/or *Aspergillus* spp., which gave initial discrepant results, but most were resolved in favour of the FBR-PCR/S result after repeat testing and clinical review (Table 2).

**Table 2:** Discrepant clinical specimens resolved in favour of molecular assay (true positive or negative by PCR/sequencing)

Specimen No.	Specimen type^a^	Stain results	Standard methods^b^	Initial molecular results	Sequence results	Results of resolution (repeat PCR and clinical review)
1	BAL LUL	CW = fungal elements	*C. albicans, A. fumigatus, A. flavus*	ITS/LSU targets POS, β-globin POS	ITS = *C. albicans* LSU = *C. albicans*	True positive PCR Fungal culture contaminated
2	BAL LLL	Gram stain = Hvy WBCs + mixed bacteria; CW = NEG	*Aspergillus spp*.	ITS/LSU targets NEG, β-globin POS	N/A	True negative PCR No pulmonary disease Fungal culture contaminated
3	BAL RLL	Gram stain – Hvy WBCs; CW = NEG	*Aspergillus spp., Penicillium spp.*	ITS/LSU targets NEG, β-globin POS	N/A	True negative PCR No pulmonary disease Fungal culture contaminated
4	BAL RUL	Gram stain − Hvy WBCs; CW = NEG	*A. fumigatus*	ITS/LSU targets NEG, β-globin POS	N/A	True negative PCR No pulmonary disease Fungal culture contaminated
5	BAL LLL	Gram stain = Hvy WBCs; CW = Hvy yeast seen	*A. fumigatus, dermatiaceous fungus*	ITS/LSU targets POS, β-globin POS	ITS = *Exophiala dermatitidis* LSU = *Exophiala dermatitidis*	True positive PCR Other BAL samples grew *A. fumigatus*, *E. jeanselmi* Interstitial pneumonia
6	BAL RLL	Gram stain = Hvy WBCs + mixed bacteria; CW = NEG	*A. fumigatus*	ITS/LSU targets POS (weak), β-globin POS	ITS = *A. fumigatus* LSU = *A. fumigatus*	True positive PCR Clinical diagnosis of pulmonary Aspergillosis
7	BAL LUL	Gram stain = Sct. WBCs + mixed bacteria; CW = NEG	*C. albicans* + oropharyngeal flora	ITS/LSU targets NEG, β-globin POS	N/A	True negative PCR *Pneumocystis jirovecii* PCR POS
8	BAL RLL	Gram stain = Hvy WBCs; CW = Hvy yeast seen	*C. albicans*, yeast not *C. albicans*	ITS/LSU targets POS, β-globin POS	Mixed sequence: ITS = *C. albicans* and *C. glabrata* LSU = *C. albicans* and *C. glabrata*	True positive PCR Consistent with oropharyngeal colonization and overgrowth of *Candida* spp.
9	BAL RLL	Gram stain = Hvy WBCs + mixed bacteria; CW = NEG	*A. niger,* *A. fumigatus*	ITS/LSU targets POS, β-globin POS	ITS = *A. niger* LSU = *A. niger*	True positive PCR Other BAL samples grew both *Aspergillus* spp. Clinical diagnosis of airway colonization
10	BAL RLL	Gram stain = Hvy WBCs + mixed bacteria; CW = NEG	*C. albicans,* *A. fumigatus*	ITS/LSU targets POS, β-globin POS	ITS = *C. albicans* LSU = *C. albicans*	True positive PCR Clinical diagnosis of metapneumovirus/enterovirus infection *C. albicans* consistent with airway colonization; *A. fumigatus* contaminant
11^c^	Brain tissue	Gram stain − Hvy WBCs; CW = NEG	No growth after 4 weeks	ITS POS LSU NEG β-globin POS	ITS: *Rhizopus oryzae* LSU: No data	True positive PCR Pathology sections positive for broad aseptate hyphae Clinical diagnosis of rhinocerebral mucormycosis
12^c^	Cheek tissue	CW = fungal elements	No growth after 6 weeks	ITS POS LSU POS β-globin POS	ITS: *Rhizopus oryzae* LSU: *Rhizopus oryzae*	True positive PCR Pathology PAS and GMS section stains showed broad aseptate hyphae Clinical diagnosis of rhinocerebral mucormycosis
13^c^	Parotid gland tissue	CW = no fungal elements	*C. albicans*	ITS POS LSU POS β-globin POS	ITS: *Rhizopus oryzae* LSU: *Rhizopus oryzae*	True positive PCR Clinical diagnosis of rhinocerebral mucormycosis
14	Sinus tract fluid	Gram stain = Hvy WBCs with mixed bacteria including yeast	*C. albicans*	ITS POS LSU POS β-globin POS	ITS: *C. glabrata* LSU: *C. glabrata*	True positive PCR MALDI-TOF MS confirmed *C. glabrata* and isolate had elevated fluconazole MIC. Fungal culture initially mis-identified
15^d^	Abdominal abscess tissue/fluid	Gram stain = Hvy WBCs + mixed bacteria; CW = NEG	No growth after 6 weeks	ITS POS LSU POS β-globin POS	ITS: *C. albicans* LSU: *C. albicans*	True positive PCR Clinical diagnosis of intra-abdominal abscess
16	Shoulder tissue (intermedullary)	Gram stain − = Hvy WBCs; CW = NEG	*Alternaria* spp.	ITS NEG LSU NEG β-globin POS	N/A	True negative PCR Clinical diagnosis of *Cutibacterium acnes* joint infection Fungal culture contaminated
17	R hip tissue	Gram stain = No WBCs; CW = NEG	Environmental fungus isolated (not further identified at reference laboratory)	ITS weak band LSU weak band β-globin POS	ITS: poor sequence LSU: poor sequence	True negative PCR No evidence of IFD Fungal culture contaminated
18^e^	Liver aspirate	Gram stain = no WBCS; CW = NEG	No growth after 4 weeks	ITS POS LSU POS β-globin POS	ITS: *Rhizomucor pusillus* LSU: *Rhizomucor pusillus*	True positive PCR Pathology sections positive for broad aseptate hyphae Clinical diagnosis of hepatosplenic mucormycosis
19^f^	Lung tissue/fluid	Gram stain = Few WBCs; CW = NEG	No growth after 6 weeks	ITS POS LSU POS β-globin POS	ITS*: Histoplasma capsulatum* LSU*:* No data	True positive PCR Pathology of lung tissue showed necrotizing granulomas with yeast morphologically consistent Clinical diagnosis of Histoplasmosis

^a^BAL samples were collected by pulmonary medicine or critical care specialists according to the Calgary Zone regional protocol. All other clinical samples were collected in the operating room or by interventional radiology under ultrasound guidance

^b^Standard methods: All isolates were recovered from fungal culture. Yeasts were identified by morphology and Vitek MS while molds were identified by morphology and conventional PCR targeted to the ITS1 and 2 gene regions

^c^Specimens 11–13 were from a previously reported case of rhinocerebral Mucomycosis due to *Rhizopus oryzae* [35] allowed optimal treatment and management

^d^Specimen 15, FBR-PCR/S results allowed appropriate management of this patient’s intra-abdominal abscesses and institution of anti-fungal therapy with cessation of broad-spectrum antibacterial agents

^e^Specimen 18 FBR-PCR/S diagnosed hepatosplenic Mucormycosis due to *Rhizomucor pusillus*, which enabled immediate appropriate anti-fungal management and drainage

^f^Specimen 19 FBR-PCR/S testing allowed for rapid confirmation of Histoplasmosis, which was also consistent with histopathology sections showing yeast with broad-based budding on Grocott’s and PAS stains

**Table 3:** Discrepant Clinical Specimens Resolved in Favour of Standard Methods (False Positive or Negative by PCR/Sequencing)^a^

Specimen No.		Specimen type^a^		Stain results		Standard methods^b^		Initial molecular results	Sequence results		Results of clinical review	
1	BAL LUL		CW = fungal elements		Negative		ITS/LSU targets POS, β-globin POS			ITS = *Oxyporus corticola* LSU = *Oxyporus corticola* Repeat testing using conventional ITS primers showed *A. terreus*		False positive PCR No pulmonary disease
2	BAL RLL		CW = no fungal elements		*C. albicans, A. flavus*		ITS/LSU targets NEG, β-globin POS (weak)			N/A		False negative PCR No pulmonary disease. Repeat testing gave same results. Likely sample deficiency
3	BAL LLL		Gram stain − Hvy WBCs; CW = NEG		*C. dublinensis, A fumigatus*		ITS/LSU targets POS; β-globin POS			Sequencing indeterminate as mixed sequences could not be resolved for accurate identification		Indeterminate PCR Mixed sequences Clinical diagnosis of Aspergillosis
4	BAL LUL		Gram stain − Hvy WBCs; CW = NEG		*C. glabrata, A. terreus*		ITS/LSU targets POS; β-globin POS			ITS = *C. glabrata* LSU = *C. glabrata*		False negative PCR Clinical diagnosis of invasive Aspergillosis with cavitary lung lesion
5	BAL		Gram stain and CW = NEG		*C. glabrata*		ITS POS (weak)/LSU target NEG, β-globin POS			ITS (short sequence) = *Fusarium merismoides*, a plant pathogen		False positive PCR Clinical diagnosis of primary lung adenocarcinoma
6	BAL RML		Gram stain = Hvy WBCs; CW = NEG		C. albicans		ITS/LSU targets NEG, β-globin POS			N/A		False negative PCR Clinical diagnosis of aspiration pneumonia. *C. albicans* consistent with airway colonization
7	CSF		Gram stain = few yeast		*Cryptococcus neoformans*		ITS NEG LSU NEG β-globin POS (weak)			N/A		False-negative PCR Repeat PCR/Sequencing Negative Likely sample deficiency given weak β-globin band
8	Bone(mandible)		Gram stain = few bacteria; CW = NEG		*C. albicans*		ITS NEG LSU NEG β-globin POS (weak)			N/A		False negative PCR Repeat PCR/Sequencing Negative Likely sample deficiency given weak β-globin band
9	Peritoneal fluid		Gram stain − Hvy WBCs; CW = NEG		*A. flavus*		ITS NEG LSU NEG β-globin POS			N/A		False negative PCR Repeat PCR/Sequencing ITS POS/LSU POS with *Aspergillus spp*. split identification
10	Dialysate fluid		Gram stain = Hvy WBCs; CW not done		*C. tropicalis*		ITS NEG LSU NEG β-globin POS			N/A		False negative PCR Repeat PCR/Sequencing Negative Fungal culture of other samples grew same organism
11	Lung tissue		Gram stain = Hvy WBCs; CW = NEG		*Coccidiodes immitis*		ITS NEG LSU NEG β-globin POS			N/A		False negative PCR Clinical diagnosis of pulmonary Coccidioidomycosis

^a^BAL samples were collected by pulmonary medicine or critical care specialists according to the Calgary Zone regional protocol. All other clinical samples were collected in the operating room or by interventional radiology under ultrasound guidance

^b^Standard methods: All isolates were recovered from fungal culture. Yeasts were identified by morphology and Vitek MS while molds were identified by morphology and conventional PCR targeted to the ITS1 and ITS 2 gene regions

FBR-PCR/S analysis made a critical difference to patient management and clinical outcome in 4 unusual cases where fungal cultures were negative (Table 2).

### Molecular assay performance

The performance of the molecular assay compared to standard methods is shown in Table 4 for BALs, and Table 5 for other clinical non-BAL specimens. The molecular assay and standard methods had similar diagnostic efficiency for both BAL (90.2%) and non-BAL specimens although both approaches had lower diagnostic efficacy (81.3%) for non-BALs.

**Table 4:** Performance of molecular assay and standard methods for bronchoalveolar lavage specimens (Clinical and Contrived)^a^

		Standard methods^**b**^		
		Positive	Negative	Total
FBR-PCR/S Assay^c^	Positive	54	0	54
	Negative	7	11	18
	Total	61	11	72
^c^Sensitivity (88.5%, 54/61), specificity (100%, 11/11), PPV (100%, 54/54), NPV (61.1%, 11/18) and efficiency 90.2% (65/72)				

		FBR-PCR/S Assay		
		Positive	Negative	Total
Standard methods^d^	Positive	54	7	61
	Negative	0	11	11
	Total	54	18	72
^d^Sensitivity (100%, 54/54), specificity (61.1%, 11/18), PPV (88.5%, 54/61), NPV (100%, 11/11) and efficiency 90.2% (65/72)				

^a^Includes 39 clinical specimens and 33 contrived specimens inoculated with a variety of fungal isolates identified by the reference lab. The molecular assay detected and accurately identified all fungal isolates in contrived BALs. *PPV* positive predictive value, *NPV* negative predictive value

^b^Standard methods: All isolates were recovered from fungal culture. Yeasts were identified by morphology and Vitek MS while molds were identified by morphology and conventional PCR targeted to the ITS1 and ITS2 gene regions

**Table 5:** Performance of molecular assay and standard methods for other types of clinical specimens (non-BALs)^a^

		Standard Methods^**b**^		
		Positive	Negative	Total
FBR-PCR/S Assay^c^	Positive	14	7	21
	Negative	7	47	54
	TOTAL	21	54	75

		FBR-PCR/S Assay		
		Positive	Negative	TOTAL
Fungal culture^c^	Positive	14	7	21
	Negative	7^a^	47	54
	Total	21	54	75
^c^Both methods had sensitivity (66.7%, 14/21), specificity (87.0%, 47/54), PPV (66.7%, 14/21), NPV (87.0%, 47/54) and efficiency 81.3% (61/75)				

^a^Includes all non-BAL clinical specimens tested. Molecular assay results were resolved by clinical review and repeat testing. 7 specimens that were FBR-PCR/S (+)/fungal culture (−) were resolved after clinical review to be true positive molecular tests and false negative cultures. See Tables 2 and 3

^b^Standard methods: All isolates were recovered from fungal culture. Yeasts were identified by morphology and Vitek MS while molds were identified by morphology and conventional PCR targeted to the ITS1 and ITS2 gene regions

Both diagnostic approaches also had similar performance in clinical specimens that showed fungal elements on microscopic examination after CW staining (Table 6). Although a negative CW stain and microscopic examination has excellent specificity and NPV, it has poor sensitivity and PPV for fungal infection. Microscopy negative BALs and other clinical specimens were negative by standard methods and FBR-PCR/S. Clinical specimens positive by microscopy (n = 10, 8.8%) demonstrated variable culture and/or PCR positivity; 6 specimens were positive by both methods, 2 were only positive by culture, and 2 were only positive by PCR. Another fifty-six (49.1%) specimens were microscopy negative but grew a variety of yeast/fungi and demonstrated variable culture and/or PCR positivity; 37 were positive by both methods, 14 were only positive by culture and 5 were only positive by PCR.

**Table 6:** Performance of molecular assay and standard methods compared to microscopy for clinical specimens including contrived BALs^a^

		CW Stain/Microscopy		
		Positive	Negative	Total
FBR-PCR/S Assay^b^	Positive	8	42	50
	Negative	2	62	64
	Total	10	104	114
^b^Sensitivity (80%, 8/10), specificity (59.6%, 62/104), PPV(16%, 8/50), NPV (96.9%, 62/64) and efficiency 61.4% (70/114)				

		CW Stain/Microscopy		
		Positive	Negative	Total
Standard methods^c^	Positive	8	51	59
	Negative	2	53	55
	Total	10	104	114
^c^Sensitivity (80%, 8/10), specificity (59.6%, 62/104), PPV (13.6%, 8/59), NPV (93.4%, 53/55) and efficiency 53.5% (61/114)				

^a^Includes the results of all BALs and clinical specimens enrolled in the study

^c^Standard methods: All isolates were recovered from fungal culture. Yeasts were identified by morphology and Vitek MS while molds were identified by morphology and conventional PCR targeted to ITS1 and ITS2 gene regions

### Implementation of the molecular assay

**Fig. 2:**
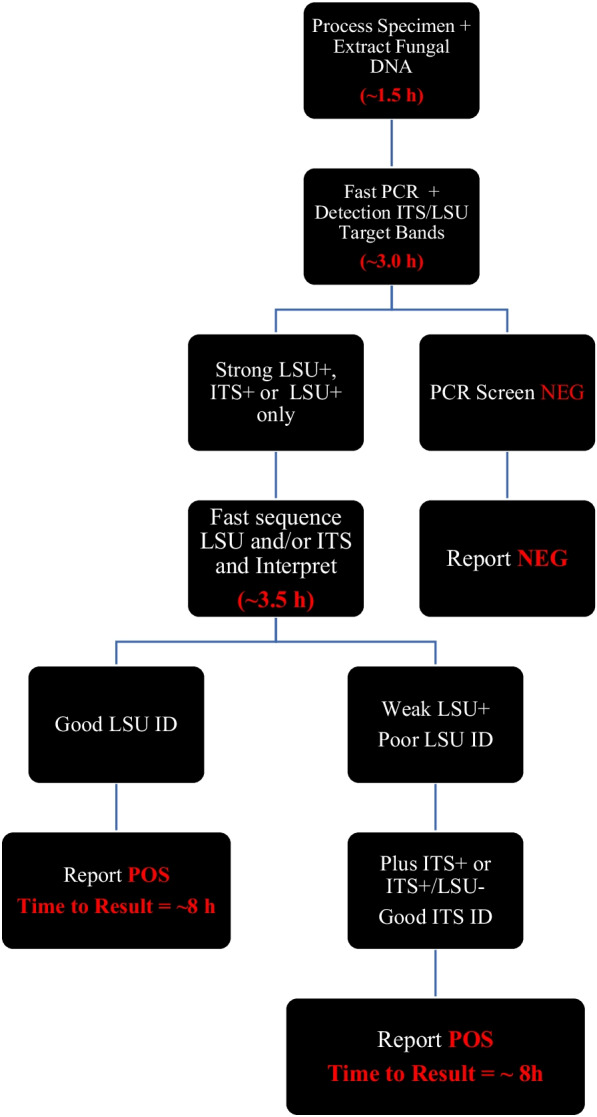
Algorithm for FBR-PCS/S Assay with Timing for Key Steps

Figure 2 shows an algorithm for the FBR-PCR/S procedure workflow and the time required for each assay step to report results in ~ 8 h or within the technologist’s dayshift; divided between specimen processing/extraction and fast PCR amplification/gel interpretation (~ 4.5 h) and fast cycle sequencing and interpretation (~ 3.5 h) (Fig. 2). Due to the longer sequence length provided by the LSU primers (> 550 bp) this would be the preferred single target for initial detection followed by ITS (~ 350 bp). To ensure an optimal pre-test probability and the quality and quantity of specimen available, FBR-PCR/S tests are ordered by the Infectious Diseases service in consultation with a medical microbiologist.

## Discussion

Our study is the first to evaluate the diagnostic performance of a novel broad-range panfungal PCR/sequencing assay using DPO primers and fast protocols in a non-selected patient population with and without confirmed, probable of possible IFD in a large Canadian health region. This approach allows equivalent or improved diagnostic performance compared to previous reports from other studies that evaluated panfungal PCR assays in both selected and non-selected patient populations and clinical specimens [19–28]. Our FBR-PCR/S assay has increased specificity compared other panfungal PCR assays and provides a rapid same day diagnosis of IFD. Our molecular assay had an excellent performance compared to culture in microscopy positive specimens, and an equivalent performance in microscopy negative specimens. IFD were diagnosed by FBR-PCR/S analysis of microscopy negative specimens indicating a role for this diagnostic approach for non-selected patients without overt immunosuppression. Rampini and colleagues (2016/Switzerland) [20] have also demonstrated similar efficacy of their fungal ITS PCR compared to conventional methods for diagnosing fungal infections in non-immunocompromised patients. They evaluated 251 clinical specimens using both the fungal ITS PCR compared to fungal culture and demonstrated a high concordance of 89.6% and equivalent analytical performance with a sensitivity, specificity, PPV and NPV of 87.7%, 90.3%, 76% and 95.5% respectively [20].

Previous reports of FBR-PCR/S evaluations in non-selected clinical cases have been limited, and primarily reported from large laboratories in Europe of the United States [19, 22, 24, 33]. Lass-Florl and colleagues (2013/Austria) [19] evaluated an ITS fungal PCR in 206 tissues and sterile fluid samples (n = 190 patients) with negative microscopy and found a sensitivity, specificity, PPV and NPV of 57.1%, 97%, 80% and 91.7%. Valero and colleagues (2016/Spain) [24] developed a fungal PCR using two ITS primers and 4 probes targeted to specific fungal pathogen groups, which showed comparable sensitivity (83.3%) and specificity (100%) to our assay. Zeller and colleagues (2017/Austria) [21] evaluated an ITS fungal PCR in 105 tissues and sterile fluids (n = 98 patients) and found a sensitivity, specificity, PPV and NPV of 87.7%, 90.3%, 76% and 95.5% respectively. Gomez and colleagues (2017/USA) [23] used a dual target (i.e., ITS 2 and D2 region of 28S) to evaluate 117 tissues and sterile fluids from 117 patients with confirmed IFD compared to 116 clinical samples from 108 patients with suspected IFD. Performance of their fungal PCR assay was better in the targeted IFD group [sensitivity (96.6%) and specificity (98.25%)] than in patients suspected of IFD [sensitivity (62.8%) and specificity (71.3%)] [22]. Ala-Houhala and colleagues (2017/Finland) [22] used a dual target ITS fungal PCR to test 37 tissue and sterile fluid specimens from 279 patients and found a sensitivity, specificity, PPV and NPV of 60.5%, 91.7%, 54.2% and 93.4% respectively. Stempak and colleagues (2019/USA) [33] also showed that fungal PCR testing had equivalent performance on analyses of 65 sterile fluid and tissue samples selected based on having all reference methods done (i.e., stains, DNA probes, culture, histopathology). However, several studies discourage the routine use of panfungal PCR testing, particularly on BALs, because no IFD cases were found that were not diagnoses by the reference methods, and due to environmental contamination the results may be difficult to interpret [25, 28, 33, 34].

Our molecular assay workflow allows same day reporting of panfungal PCR results allowing for rapid diagnosis and prompt implementation of appropriate management. Use of our panfungal assay improved clinical management and outcomes for several critically ill patients whose prior work-up by standard methods had been repeatedly negative or was delayed due to the extended incubation required of fungal culture isolate recovery. Clinical laboratories may also provide a similarly rapid 16S broad-range PCT/sequencing result (~ 8 h) by implementing molecular assays based on DPO primers for both bacteria and fungal pathogens with our recommended integrated workflow.

Our study had several limitations including the small number of specimens across the various types and sources enrolled. Because *Candida* spp. and *Aspergillus* spp. are the most commonly isolated fungi from BAL and non-BAL specimens in clinical microbiology laboratories worldwide, we used contrived BAL specimens to broaden the evaluation of the FBR-PCR/S assay. Due to the inherently high rate of contamination by fungal commensals present in clinical samples, interpretation of both standard methods compared to panfungal PCR results may be challenging without clinical review as shown by the initial rate of discordant results in this study. BALs or other pulmonary samples were most contaminated by commensal fungi in the patient’s airway, particularly *Candida* spp., *Penicillium* spp. and *Aspergillus* spp., which occurs during collection. But as previously reported [25, 28, 34], this problem is not unique to our study. Panfungal PCR alone may also not be optimal for diagnosing polymicrobial fungal infections because mixed sequencing results may not be interpretable. A more detailed clinical assessment by chart review would have allowed a more accurate clinical assessment for the presence of IFD.

## Conclusions

Rapid panfungal FBR-PCR/S testing has equivalent diagnostic efficiency compared to standard methods with improved specificity, but our novel assay improves clinical utility by reporting a rapid species-level identification the same dayshift (~ 8 h).

## Data Availability

The data that support the findings of this study are available from Alberta Health Services (AHS), Alberta Precision Laboratories (APL) (formerly CLS) but restrictions apply to the availability of these data, which were used under the ethics agreement for the current study, and so are not publicly available. Data are however available from the author upon reasonable request and with permission of AHS/APL.

## Abbreviations

FBR-PCR/S: Fast broad range PCR and sequencing
PCR: Polymerase chain reaction
BAL: Bronchoalveolar lavage
CS: Clinical specimens
CSF: Cerebrospinal fluid
IFD: Invasive fungal diseases
CW: Calcofluor white stain
ITS: Internal transcribed spacer region
LSU: Large subunit region
DNA: Deoxyribonucleic acid
NEC: Negative extraction control
PEC: Positive extraction control
DPO: Dual priming oligonucleotides
IMA: Inhibitory mold agar
BHI: Brain heart infusion agar
BHIA: Brain heart infusion agar with antibiotics
BCYE: Buffered charcoal yeast extract agar
HIV/AIDS: Human immunodeficiency virus/acquired immunodeficiency syndrome
HSCT: Hematopoietic stem cell transplant
SOT: Solid organ tumour
PPV: Positive predictive value
NPV: Negative predictive value
PEC: Positive extraction control
